# The impact of social participation on activities of daily living in older adults with knee osteoarthritis: the chain mediating effects of sleep quality and multidimensional frailty

**DOI:** 10.3389/fpsyg.2025.1745738

**Published:** 2026-02-02

**Authors:** Huiqiong Tu, Tingting Zhao, Lan Chen, Yanyan Ling, Yelin Zhang, Jianling Wei, Xiaomeng Liang, Li Zhou, Yushi Liu

**Affiliations:** 1 Ruikang Hospital Affiliated to Guangxi University of Chinese Medicine, Nanning, China; 2 School of Nursing, Guangxi University of Chinese Medicine, Nanning, China

**Keywords:** activities of daily living, knee osteoarthritis, multidimensional frailty, sleep quality, social participation

## Abstract

**Introduction:**

This study aimed to investigate the associations among social participation, sleep quality, multidimensional frailty, and activities of daily living (ADL) in older adults with knee osteoarthritis (KOA), and to further evaluate whether sleep quality and frailty jointly mediate these relationships.

**Methods:**

A cross-sectional study was conducted among 288 older adults with KOA recruited via convenience sampling. Validated scales were used to assess social participation, sleep quality, multidimensional frailty, and ADL. Statistical analyses were performed using SPSS 26.0 and the PROCESS 4.1 macro, including descriptive statistics, correlation analyses, and mediation modeling.

**Results:**

Social participation was significantly associated with sleep quality, multidimensional frailty, and ADL (all *p* < 0.001). Sleep quality was significantly associated with frailty and ADL, and frailty was also associated with ADL. Mediation analysis indicated that sleep quality and frailty each partially mediated the association between social participation and ADL, and jointly formed a significant chain mediation pathway. The combined indirect effect was 0.315, accounting for 42.51% of the total effect.

**Discussion:**

Social participation was significantly associated with ADL in older adults with KOA, both directly and indirectly through sleep quality and multidimensional frailty. These findings suggest that enhancing social engagement and addressing sleep and frailty issues may be important for maintaining functional independence in this population. Future longitudinal and interventional studies are needed to validate these findings and inform targeted strategies for improving daily functioning among older adults with KOA.

## Introduction

Knee osteoarthritis (KOA) is a highly prevalent degenerative joint condition affecting over 250 million people worldwide, and its burden is increasing rapidly with population aging (Garstang and Stitik, 2006; Lee et al., 2019; Chen et al., 2024). Older adults living with KOA commonly experience persistent pain, stiffness, and mobility limitations (Cheng et al., 2012; Zhang et al., 2020), which are strongly associated with declines in functional independence and overall quality of life (Clynes et al., 2019). In addition, movement limitations related to KOA may complicate the management of coexisting chronic conditions (e.g., dyslipidemia or metabolic disorders), potentially leading to increased healthcare utilization and costs (Lv et al., 2025). These symptoms often interfere with the ability to perform activities of daily living (ADL) and contribute to broader challenges in maintaining physical, psychological, and social well-being (Wojcieszek et al., 2022). Given the growing number of older adults affected and the close link between KOA symptoms and functional impairment, it is essential to better understand the factors associated with ADL among this population.

Social participation—defined as engagement in social, community, or interpersonal activities—is a key component of active and healthy aging (Dehi Aroogh and Mohammadi Shahboulaghi, 2020). Higher levels of social engagement have been associated with better emotional well-being, stronger social networks, greater physical activity, and improved quality of life in older adults (Parra-Rizo, 2020; Parra-Rizo et al., 2024). Older adults with KOA often experience reduced mobility and pain-related avoidance of social activities, which is frequently associated with lower levels of social interaction and increasing isolation (Santini et al., 2016; Vaughan et al., 2017; Siviero et al., 2020; Gao et al., 2023; Tsoli et al., 2024). Prior studies have shown that limited social participation is linked to poorer physical functioning and reduced independence in daily life among older adults (Philip et al., 2020; Zhou et al., 2025). However, the pathways linking social participation with functional outcomes in older adults with KOA remain unclear, underscoring the need to investigate how social engagement may be related to ADL in this population.

Sleep quality is a fundamental determinant of overall quality of life and plays a critical role in physical and mental well-being, influencing chronic disease risk, symptom burden, and disease management (Lu et al., 2025). Sleep quality is closely linked to health and daily functioning in older adults with KOA (Jacob et al., 2021). Inadequate sleep is generally associated with poorer physical and psychological outcomes, including greater limitations in ADL, whereas adequate sleep corresponds to better functional status (Webb et al., 2018; Denison et al., 2021; Yang et al., 2023). Lower social participation has also been related to poorer sleep quality, suggesting that sleep may serve as an intermediary through which social engagement influences daily functioning (Chen et al., 2016; Rawtaer et al., 2018; Wang Y. et al., 2021). As sleep disturbances often co-occur with increased vulnerability across physical, psychological, and social domains, examining sleep quality is essential for understanding broader pathways that may contribute to multidimensional frailty.

Multidimensional frailty, which reflects vulnerability across physical, psychological, and social domains (Gobbens et al., 2010a), has been consistently associated with greater dependence in ADL (van der Vorst et al., 2018). Lower social participation has also been linked to higher levels of frailty, suggesting a close interplay among social functioning, vulnerability, and daily independence (Fang et al., 2022; Saeki et al., 2023). However, although frailty has been linked to both social participation and ADL, it remains unclear whether frailty helps explain these associations in older adults with KOA. Evidence examining the mediating role of frailty in this population is still limited.

Evidence has shown that sleep disturbances are closely associated with multidimensional frailty. A Chinese study reported that insomnia was independently related to higher frailty levels (Fan et al., 2022), and a recent systematic review further confirmed a positive association between sleep problems and frailty among community-dwelling older adults (Qi et al., 2025). Wang et al. (2024) also found that sleep quality played a mediating role in the association between social participation and frailty within a chain mediation model. However, these studies have not incorporated ADL as a functional outcome, nor have they focused on high-risk clinical populations such as older adults with KOA. Addressing these gaps, the present study extends existing evidence by examining whether sleep quality and multidimensional frailty jointly mediate the association between social participation and ADL in this vulnerable population.

Guided by the Active Aging Framework—which highlights social participation, physical health, psychological well-being, and quality of life as core components of healthy aging—this study conceptualizes social participation as the initiating factor in the proposed model (Kalache and Gatti, 2003). Social engagement has been associated with better emotional well-being, healthier behaviors, and greater functional autonomy, suggesting its potential upstream influence on sleep quality, multidimensional frailty, and daily functioning in later life (Bar-Tur, 2021). Although previous studies have separately linked social participation to sleep disturbances (Endeshaw and Yoo, 2019), frailty (Sun et al., 2022), or functional limitations (Wen et al., 2024), little is known about how these factors operate together among older adults with KOA. Prior research has neither incorporated ADL as a key functional outcome nor examined whether sleep quality and frailty may act as sequential mediators in this high-risk group. Building on this evidence, the present study proposes that social participation may be related to ADL via sleep quality and multidimensional frailty within a chain mediation pathway.

Accordingly, the following hypotheses are proposed:

*H1*: Sleep quality mediates the relationship between social participation and ADL in older adults with KOA.

*H2*: Multidimensional frailty mediates the relationship between social participation and ADL in older adults with KOA.

*H3*: Sleep quality and multidimensional frailty chain-mediate the relationship between social participation and ADL in older adults with KOA.

**Figure 1:**
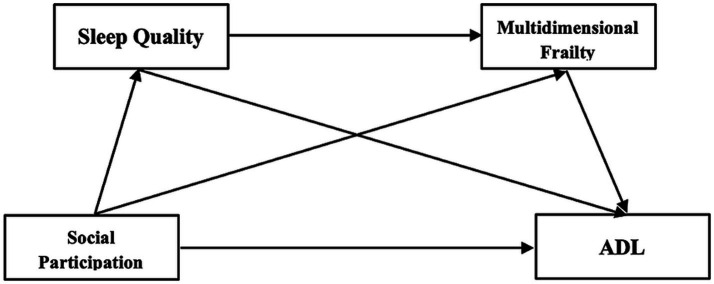
Research hypothesis model the proposed theoretical model assumes that social participation is the initiating factor that affects both frailty and ADL, directly and indirectly through sleep quality. This assumption is grounded in the Active Aging Framework, which emphasizes social participation as a key determinant of health, functioning, and well-being in later life (Niu et al., 2016). According to this framework, social engagement fosters positive health behaviors, emotional resilience, and cognitive functioning, all of which are protective against frailty and functional decline (Lv et al., 2025).

This study uniquely examines these mediating pathways by integrating ADL as a functional outcome and applying the model to a KOA population, offering new insights into social and health mechanisms that shape independence in later life. The theoretical model is presented in Figure 1.

## Methods

### Study design

This study adopted a cross-sectional design to examine the associations among social participation, sleep quality, multidimensional frailty, and ADL in older adults with KOA. The reporting of this study adheres to the STROBE guidelines, ensuring completeness and transparency in methodological description. A cross-sectional approach was chosen because it allows efficient assessment of interrelationships among multiple psychosocial and functional variables within a relatively short time frame. However, we acknowledge that this design limits causal inference, and longitudinal studies are needed to confirm temporal pathways.

### Participant recruitment and sampling

Participants were recruited using a convenience sampling approach from the orthopedic outpatient and inpatient departments of Ruikang Hospital between January and April 2025. Convenience sampling was adopted because KOA patients regularly attend follow-up visits in this clinical setting, making it feasible to enroll an adequate sample within the study period. To reduce selection bias, participants were approached consecutively as they became eligible.

A total of 326 older adults with KOA were screened, of whom 22 did not meet the inclusion criteria and 16 declined participation due to limited time, discomfort with questionnaire completion, or reluctance to share personal information. The final sample comprised 288 participants.

### Eligibility criteria

Participants were eligible for inclusion if they met all of the following criteria:

#### Inclusion criteria

1. Age ≥60 years, ensuring the sample represents the older adult population.
2. Clinical diagnosis of KOA according to the 2019 Chinese guidelines (Zhang et al., 2020).
3. Fully conscious, defined as intact orientation to person, place, and time, and the ability to understand study instructions.
4. Normal communication abilities, meaning they could clearly express responses without cognitive or sensory impairments.
5. No history of psychiatric disorders that might affect self-report accuracy.
6. Provided written informed consent voluntarily.

#### Exclusion criteria

1. Total knee arthroplasty within the past 6 months, as postoperative recovery may directly affect physical function, sleep, frailty, and ADL.
2. Severe comorbidities or unstable vital signs, which could interfere with participation or bias functional assessments.
3. Physiotherapy or rehabilitation within the last 6 months, since recent interventions may alter frailty level, sleep quality, or social participation and introduce confounding effects.

### Sample size calculation

The sample size was estimated using the commonly applied formula for multivariable analyses:


N≥m×(5–10)

where *m* is the number of observed variables (Ni et al., 2010). With 24 variables, the required sample size ranged from 120 to 240. To ensure adequate model stability and reduce the risk of overfitting, we adopted the upper bound (10 participants per variable), yielding a minimum target of 240 participants. The final sample of 288 exceeded this requirement. A formal power analysis was not feasible due to the lack of prior effect-size estimates, making the variable-based rule of thumb a suitable conservative alternative.

### Data collection procedures

Data were collected through structured face-to-face interviews conducted by three trained investigators. All investigators received unified training on questionnaire instructions, standardized explanations, and non-directive communication to ensure consistency and reliability. To minimize response bias, investigators followed a written script, avoided leading prompts, and provided only neutral clarification. All questionnaires were checked immediately after completion. Any missing or unclear responses were resolved on-site with participants before final data entry.

Additional clinical variables such as pain intensity, radiographic severity, depressive or anxiety symptoms, physical activity level, medication use, and detailed comorbidity profiles were not collected because incorporating these assessments into routine clinical workflows would have substantially increased participant burden and reduced feasibility.

### Human ethics and consent to participate declarations

This study was approved by the Ethics Committee of Ruikang Hospital Affiliated to Guangxi University of Chinese Medicine (Approval No. KY2024-185). All participants provided written informed consent after being informed of the study purpose, procedures, voluntary nature of participation, and their right to withdraw at any time. To ensure confidentiality, all questionnaires were anonymized using unique identification codes, and no personally identifiable information was stored. Data were kept in password-protected files accessible only to the research team, and all records were stored securely and used solely for research purposes.

### Research tools

#### Sociodemographic characteristics

For this study, the questionnaire was formulated by the research team after reviewing and integrating findings from relevant studies and is divided into two main categories: demographic characteristics and clinical information. Demographic data covered variables such as gender, age, place of residence, marital status, and educational background. Clinical information included details on disease duration, affected sites, and coexisting conditions.

#### Activities of daily living

The Activities of Daily Living Scale, originally developed by Katz et al. (1963), assesses functional independence in older adults through both Basic Activities of Daily Living (BADL) and Instrumental Activities of Daily Living (IADL) (Lawton and Brody, 1969). This tool is widely used in musculoskeletal and geriatric research and is particularly suitable for older adults with KOA because pain, stiffness, and mobility limitations directly influence their capacity to perform daily self-care and household activities (Nanjo et al., 2023; Sundaram et al., 2023). Numerous studies have validated its use in KOA populations in China, demonstrating strong applicability and clinical relevance (Wang et al., 2021b; Yan et al., 2024). The scale includes six BADL items and eight IADL items, each rated on a 4-point Likert scale (1 = no difficulty to 4 = unable to perform). Total scores range from 14 to 56, with higher scores indicating more severe ADL impairment. Consistent with prior research, levels of impairment are categorized as normal (<14), mild (15–22), and severe (>22). In the present study, the scale demonstrated good internal consistency (Cronbach’s *α* = 0.867).

#### Social participation

Social participation was measured using the Social Dysfunction Screening Scale derived from the World Health Organization Disability Assessment Schedule (WHO-DAS) (World Health Organization and Pan American Health, 1988). The 10-item scale evaluates engagement in key social roles, including productive activities, household responsibilities, social interactions, community involvement, and self-care. It is appropriate for older adults with KOA, as pain and mobility limitations commonly restrict their ability to participate in daily and social activities. Each item is rated from 0 to 2, yielding a total score of 0–20, with higher scores indicating poorer social participation. Scores ≥2 suggest impaired participation. The scale has been widely applied in Chinese studies involving older adults with chronic or musculoskeletal conditions and has demonstrated good psychometric properties (Tao et al., 2020; Wang et al., 2021a; Zhu et al., 2023; Sun et al., 2024). In this study, internal consistency was acceptable (Cronbach’s *α* = 0.780).

#### Sleep quality

Sleep quality was assessed using the Pittsburgh Sleep Quality Index (PSQI), developed by Buysse et al. (1989). The PSQI evaluates sleep disturbances across seven domains, including subjective sleep quality, sleep latency, sleep duration, sleep efficiency, sleep disturbances, use of sleep medication, and daytime dysfunction. Sleep problems are highly prevalent among older adults with KOA, as pain, limited mobility, and nocturnal discomfort often disrupt sleep patterns, making the PSQI particularly suitable for this population. The instrument consists of 18 items generating a global score ranging from 0 to 21, with higher scores indicating poorer sleep quality. A commonly adopted cut-off in China classifies scores ≥7 as poor sleep (Tsai et al., 2005). The Chinese version of the PSQI has demonstrated sound reliability and validity in older adults and has been widely used in studies involving KOA populations in China, supporting its applicability in this clinical group (Chen et al., 2015; Lü et al., 2017; Ran et al., 2021). In the present study, internal consistency was good (Cronbach’s *α* = 0.864).

#### Multidimensional frailty

Multidimensional frailty was assessed using the Tilburg Frailty Indicator (TFI), which was developed by Gobbens et al. (2010b) based on the multidimensional model of frailty that incorporates physical, psychological, and social components. The TFI is particularly suitable for older adults with KOA, as chronic pain, limited mobility, psychological burden, and social withdrawal commonly contribute to vulnerability across these three domains. The instrument contains 15 items, including 8 physical, 4 psychological, and 3 social items. Most items are scored dichotomously, while items 9, 10, 11, and 14 use a three-level scoring format. Total scores range from 0 to 15, with higher scores indicating greater frailty; values ≥5 are commonly used to classify individuals as frail. The Chinese version, translated and validated by Dong et al. (2017), has demonstrated satisfactory psychometric properties and has been widely applied in studies involving older adults in China, including those with chronic musculoskeletal conditions (Xie et al., 2020; Fan et al., 2022; Xue et al., 2024). In the present study, internal consistency was acceptable (Cronbach’s α = 0.702).

#### Operationalization of variables

All four scales in this study use the same scoring direction: higher scores indicate worse outcomes. To ensure clarity, the interpretation for each variable is summarized below:

1. Social Participation: Score range 0–20; higher scores = poorer participation; scores ≥2 indicate impairment.
2. PSQI: Range 0–21; higher scores = poorer sleep; ≥7 denotes poor sleep.
3. TFI: Range 0–15; higher scores = greater frailty; ≥5 indicates frailty.
4. ADL: Range 14–56; higher scores = greater impairment; classified as normal (<14), mild (15–22), and severe (>22).

These thresholds were consistently applied in all analyses to ensure accurate interpretation of variable direction.

### Statistical analysis

Statistical analyses were conducted using SPSS version 26.0 (IBM Corp., Armonk, NY, USA) and the PROCESS macro version 4.1. Continuous variables were first examined for normality using the Kolmogorov–Smirnov test. Normally distributed data are presented as mean ± standard deviation (SD), whereas non-normally distributed variables are summarized as medians with interquartile ranges (IQR). Categorical variables are reported as frequencies and percentages.

Correlation analyses were performed using Pearson’s correlation for normally distributed variables and Spearman’s rank correlation for non-normally distributed variables. To examine the hypothesized mediation pathways, a chain mediation model (PROCESS Model 6) was applied, with social participation as the independent variable, ADL as the dependent variable, and sleep quality and multidimensional frailty as sequential mediators. Bias-corrected bootstrap confidence intervals (5,000 resamples, 95% CI) were used to estimate indirect effects. A two-tailed *p*-value < 0.05 was considered statistically significant.

Missing data were minimal (<1%) and were addressed on-site during data collection, with investigators verifying incomplete responses immediately; therefore, no additional imputation procedures were required.

## Results

Table 1 summarizes the demographic and clinical characteristics of the 288 participants diagnosed with KOA.

**Table 1:** Basic information of survey respondents (*n* = 288).

Variables	*M (SD)* or *N* (%)
Age (years)	69.90 ± 6.66
Sex	
Male	111 (38.5%)
Female	177 (61.5%)
Residence	
Urban	187 (64.9%)
Suburban	57 (19.8%)
Rural	44 (15.3%)
Marital status	
Married	215 (74.7%)
Divorced/widowed	58 (20.1%)
Single	15 (5.2%)
Education level	
Primary or below	71 (24.7%)
Middle school	101 (35.0%)
High school/technical school	77 (26.7%)
College or above	39 (13.6%)
Disease duration	
<1 year	42 (14.6%)
1–5 years	93 (32.3%)
6–10 years	72 (25.0%)
>10 years	81 (28.1%)
Affected area	
Single knee	132 (45.8%)
Both knees	156 (54.2%)
Comorbidities	
Yes	184 (63.9%)
No	104 (36.1%)

M, mean; SD, standard deviation; N, absolute frequency; %, relative frequency. Comorbidities refer to physician-diagnosed chronic diseases including hypertension, diabetes, cardiovascular disease, chronic respiratory disease, and others.

### Common method bias test

To examine the presence of common method bias, Harman’s single-factor analysis was performed on the 63 items measuring ADL, social participation, sleep quality, and multidimensional frailty. The unrotated principal component analysis yielded 12 factors with eigenvalues above 1. The first factor explained 23.83% of the variance, which is below the 40% criterion, and the cumulative variance explained by all 12 factors was 62.84%. These results suggest that common method bias is not a significant concern in the study data.

### Social participation, sleep quality, multidimensional frailty, and ADL levels in older adults with KOA

Older adults with KOA in this study had a median social participation score of 10 (IQR: 7–12), indicating low levels of social participation (scores ≥2). The median sleep quality score was 7 (IQR: 6–11), reflecting poor sleep quality (scores ≥7). The median multidimensional frailty score was 7 (IQR: 4–9), with scores ≥5 suggesting frailty across physical, psychological, and social domains. The median ADL score was 17 (IQR: 14–23), indicating mild to moderate impairment in daily functioning (scores 15–22 indicate mild impairment, >22 severe impairment).

### Correlations among social participation, sleep quality, multidimensional frailty, and ADL in older adults with KOA

The correlation matrix in Table 2 shows that lower social participation was significantly associated with poorer sleep quality (*r* = 0.663, *p* < 0.01), greater frailty (*r* = 0.540, *p* < 0.01), and more severe ADL impairment (*r* = 0.663, *p* < 0.01). Similarly, worse sleep quality was associated with greater frailty (*r* = 0.491, *p* < 0.01) and higher ADL impairment (*r* = 0.504, *p* < 0.01). In addition, higher frailty was significantly correlated with greater impairment in ADL (*r* = 0.579, *p* < 0.01).

**Table 2:** Correlation analysis results of each variable (*N* = 288).

Variable	Social participation	Sleep quality	Multidimensional frailty	ADL
Social participation	1.000			
Sleep quality	0.663***	1.000		
Multidimensional Frailty	0.540***	0.491***	1.000	
ADL	0.663***	0.504***	0.579***	1.000

*p < 0.05, **p < 0.01, ***p < 0.001.

Higher scores on the social participation, sleep quality, frailty, and ADL scales indicate greater social dysfunction, poorer sleep quality, higher levels of frailty, and more severe ADL impairment, respectively.

### Mediation analyses

Before conducting the mediation analysis, multicollinearity among the predictors and mediators was assessed. All variables showed acceptable tolerance values (0.55–0.71) and low VIF values (1.40–1.82), indicating that multicollinearity was not a concern.

Using Model 6 from PROCESS version 4.1 and controlling for demographic variables (gender, age, residence, marital status, and education level)—which were included because these factors are known to influence social participation, sleep quality, frailty, and ADL in older adults (Cheng et al., 2022; Yang et al., 2022; Keshari and Shankar, 2023; Liu et al., 2024)—a chain mediation analysis was conducted with social participation (X) as the independent variable, ADL (Y) as the dependent variable, and sleep quality (M1) and multidimensional frailty (M2) as mediators.

**Figure 2:**
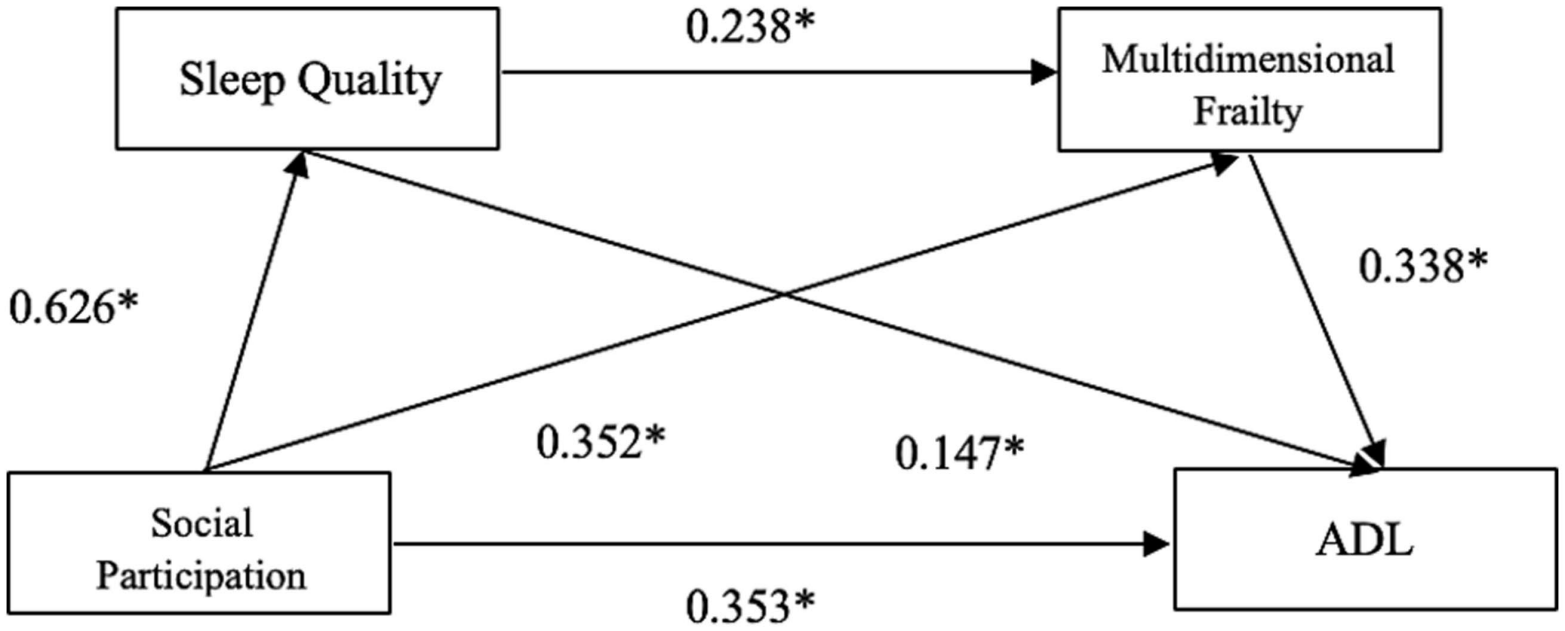
Chain mediation model linking social participation to ADL via sleep quality and multidimensional frailty in older adults with KOA.

The results indicated that:

1. Lower social participation (i.e., higher scores on the social participation scale) was significantly associated with poorer sleep quality, with an unstandardized coefficient of *β* = 0.689 (*p* < 0.01) and a standardized coefficient of *β* = 0.626.
2. Both lower social participation greater social dysfunction (*β* = 0.264, standardized *β* = 0.352, *p* < 0.01) and poorer sleep quality (*β* = 0.162, standardized *β* = 0.238, *p* < 0.01) were significantly associated with higher levels of multidimensional frailty.
3. When ADL was the dependent variable, greater social dysfunction (*β* = 0.426, standardized *β* = 0.353, *p* < 0.01), poorer sleep quality (*β* = 0.160, standardized *β* = 0.147, *p* < 0.01), and higher frailty (*β* = 0.543, standardized *β* = 0.338, *p* < 0.01) were all significantly associated with more severe impairment in ADL (see Figure 2).

See Table 3 for detailed results.

**Table 3:** Results of the chain mediation model linking social participation to ADL via sleep quality and multidimensional frailty (*N* = 288).

Dependent variable	Independent variable	*R*^2^	*F*	*β* (unstandardized)	*β* (standardized)	SE	*t*	*P*
Sleep quality	Social participation	0.391	184.135	0.689	0.626	0.050	13.569	<0.01
Multidimensional frailty	Social participation	0.285	57.010	0.264	0.352	0.048	5.484	<0.01
	Sleep quality			0.162	0.238	0.043	3.713	<0.01
ADL	Social participation	0.490	91.171	0.426	0.353	0.068	6.186	<0.01
	Sleep quality			0.160	0.147	0.061	2.637	<0.01
	Multidimensional frailty			0.543	0.338	0.080	6.747	<0.01

Higher scores on the social participation, sleep quality, frailty, and ADL scales indicate greater social dysfunction, poorer sleep quality, higher levels of frailty, and more severe ADL impairment, respectively.

Table 4 summarizes the total, direct, and indirect effects. The bootstrap confidence intervals for all three mediation paths did not cross zero, confirming the robustness of the proposed chain mediation model. The overall impact of social participation on ADL was 0.741, including a direct effect of 0.426 (57.49% of the total) and an indirect effect of 0.315 (42.51%).

**Table 4:** Indirect, direct, and total effects in the chain mediation model of social participation, sleep quality, frailty, and ADL (*N* = 288).

Effect	Effect value	Standard error	95% CI	Proportion of total effect (%)
Total effect	0.741	0.056	0.631 ~ 0.852	100
Direct effect	0.426	0.068	0.290 ~ 0.562	57.49
Total indirect effect	0.315	0.050	0.222 ~ 0.419	42.51
Social participation → Sleep quality → ADL	0.110	0.043	0.030 ~ 0.200	14.93
Social participation → Multidimensional frailty → ADL	0.143	0.034	0.081 ~ 0.217	19.37
Social participation → Sleep quality → Multidimensional frailty → ADL	0.060	0.018	0.026 ~ 0.100	8.21

Higher scores on the social participation, sleep quality, frailty, and ADL scales indicate greater social dysfunction, poorer sleep quality, higher levels of frailty, and more severe ADL impairment, respectively.

The total indirect effect was further composed of three significant pathways:

- Through sleep quality alone (0.110), representing 14.93% of the total effect;
- Through multidimensional frailty alone (0.143), accounting for 19.37%;
- Through the sequential path via sleep quality and frailty (0.060), comprising 8.21%.

These findings demonstrate that both direct and indirect pathways play substantial roles in the relationship between social participation and ADL.

### Summary of main findings

This study found that lower social participation was associated with poorer sleep quality, greater multidimensional frailty, and higher ADL impairment in older adults with KOA. Both sleep quality and frailty independently mediated these associations, and they also formed a sequential chain pathway. To our knowledge, this is the first study to show that sleep quality and multidimensional frailty jointly explain the link between social participation and ADL in this population.

### Interpretation of key associations

The correlation patterns revealed that social participation had the strongest association with ADL (*r* = 0.663), suggesting that reduced engagement in social activities is closely linked to greater functional impairment in older adults with KOA. This finding supports previous evidence indicating that maintaining social involvement helps preserve daily functional capacity (Li and Wu, 2022; Yan et al., 2024). Sleep quality also showed meaningful associations with frailty (*r* = 0.491) and ADL impairment (*r* = 0.504). These relationships are consistent with literature demonstrating that poor sleep contributes to fatigue, decreased cognitive alertness, and reduced physical performance, which collectively hinder the ability to carry out daily activities (Whibley et al., 2019; Yang et al., 2023). Frailty was moderately correlated with ADL (*r* = 0.579), reinforcing the well-established understanding that physiological, psychological, and social vulnerability substantially increases the likelihood of functional decline (van der Vorst et al., 2018; Zhao et al., 2024). Taken together, these results illustrate an interconnected pattern in which social disengagement, sleep disturbances, and frailty coexist and collectively shape daily functioning in older adults with KOA.

## Mechanisms

### Mediating Role of Sleep Quality

Sleep quality emerged as a significant mediator between social participation and ADL, indicating that reduced social engagement may indirectly contribute to functional decline through its impact on sleep. While previous studies have linked low social participation to psychological distress and poor sleep (Chen et al., 2016; Rawtaer et al., 2018; Wang et al., 2021c), and separately associated sleep disturbances with impaired physical and cognitive functioning (Webb et al., 2018; Denison et al., 2021; Yang et al., 2023), these pathways have rarely been examined together. By integrating these associations, the present study extends existing work and demonstrates that poor sleep may reduce emotional stability, heighten inflammation and pain sensitivity, and diminish daytime energy (Spira et al., 2009; Quartana et al., 2015; Hu et al., 2020; Shi et al., 2020; Kuna et al., 2022; Hertel et al., 2023), thereby limiting the capacity to perform daily tasks. These findings highlight sleep quality as a key psychological–physiological mechanism through which social participation influences functional outcomes in KOA.

### Mediating Role of Multidimensional Frailty

Multidimensional frailty also mediated the association between social participation and ADL. Limited social engagement can weaken physical activity levels, increase emotional distress, and reduce access to social resources, thereby contributing to frailty across physical, psychological, and social domains. Physically, reduced activity accelerates functional decline and loss of muscle strength (Xie and Ma, 2021). Psychologically, lower engagement heightens loneliness and stress, which are recognized drivers of frailty progression (Levasseur et al., 2017). Socially, fewer opportunities for interpersonal interaction diminish support and coping capacity (Provencher et al., 2016; Anjum et al., 2018). These multidomain deficits collectively increase the risk of ADL impairment, consistent with previous findings that frailty strongly predicts functional decline (van der Vorst et al., 2018). By demonstrating frailty’s mediating role specifically in a KOA population, this study extends earlier research and highlights frailty as a central mechanism through which social participation influences daily functioning.

### Sequential Chain Mediation: Sleep Quality and Frailty

This study also identified a sequential pathway whereby reduced social participation affected ADL through its impact on sleep quality and multidimensional frailty. Limited social engagement can heighten loneliness and psychological stress, leading to sleep disturbances in older adults (Zhao et al., 2022). Poor sleep then produces a cascade of physiological and functional consequences—including impaired inflammatory regulation (Medic et al., 2017), weakened immune and cardiovascular function (Lao et al., 2018; Kuna et al., 2022), reduced cognitive performance (Niu et al., 2016), and lower daytime energy (Balomenos et al., 2021)—all of which increase vulnerability to frailty. As frailty progresses, declines in physical reserves, psychological resilience, and social functioning further restrict mobility and confidence, reinforcing functional dependence and worsening ADL performance (Bunt et al., 2017; Esbrí-Víctor et al., 2017; Zhang et al., 2018). This aligns with prior findings showing that sleep problems accelerate frailty development (Wang et al., 2024), but our results extend existing evidence by demonstrating the full sequential mechanism in a KOA population. To our knowledge, this is the first study to show that sleep disturbance and multidimensional frailty jointly mediate the association between social participation and ADL, underscoring the need for integrated interventions that target social engagement, sleep health, and frailty prevention.

### Implications for Practice

This study highlights several practical implications for the care of older adults with KOA. Enhancing social participation may help prevent declines in sleep quality, frailty, and ultimately ADL performance, suggesting that community engagement programs and group-based activities should be integrated into routine management (Levy et al., 2020). The mediating role of sleep quality underscores the need for regular sleep assessment and targeted interventions such as sleep hygiene education and pain management (Gupta et al., 2023). In addition, because frailty contributes substantially to functional impairment, multidomain interventions addressing physical, psychological, and social vulnerability may be beneficial (Dedeyne et al., 2017). Overall, promoting social participation and improving sleep quality may serve as key strategies to preserve functional independence in this population.

### Strengths/limitations

This study has several notable strengths. It simultaneously examined social participation, sleep quality, multidimensional frailty, and ADL within a single analytic framework, allowing a more integrated understanding of functional decline in older adults with KOA. By testing a chain mediation model, the study provides new evidence regarding the sequential pathways linking psychosocial and physical factors—an approach not previously applied to this population. In addition, all measurements were obtained using validated instruments with good reliability, enhancing the robustness of the findings. Several limitations should also be acknowledged, as they may influence the interpretation of the findings. First, the cross-sectional design restricts causal inference, meaning that the temporal ordering among social participation, sleep quality, frailty, and ADL cannot be firmly established. Second, the use of self-reported questionnaires introduces potential recall and reporting bias, which may affect the accuracy of measurements, particularly for sleep quality, frailty, and ADL. Third, several important health-related confounders were not collected, including depression, pain intensity, medication use, physical activity, and comorbidity profiles. These variables were not included because the study was conducted within routine clinical workflows, where adding additional assessments would substantially increase participant burden and reduce feasibility. Given their known influence on sleep, frailty, social functioning, and daily activities, their omission may have introduced residual confounding and may partly over- or underestimate the mediation pathways observed in this study. Fourth, radiographic severity (e.g., KL grade) was not assessed, preventing evaluation of whether the studied associations differ across KOA severity levels. Fifth, the gender imbalance in the sample may limit generalizability, as men and women differ in social engagement patterns, vulnerability to frailty, and sleep characteristics. Finally, although the sample size met conventional variable-based criteria, a formal power analysis was not conducted, which may limit the precision of effect estimates.

### Future Research

Future studies should adopt longitudinal or interventional designs to clarify the temporal pathways linking social participation, sleep quality, frailty, and ADL. Incorporating key health-related confounders—such as pain intensity, depressive symptoms, medication use, physical activity, and comorbidity profiles—will help reduce residual bias and refine mediation estimates. Including radiographic severity indicators (e.g., KL grade) and recruiting samples with more balanced gender distributions will improve generalizability. Larger, multicenter studies using formal power calculations are also recommended. In addition, exploring other potential mediators or moderators, such as psychological resilience, social support, or inflammatory markers, may further deepen understanding of how social participation affects functional outcomes in older adults with KOA.

## Conclusion

This study clarifies how social participation, sleep quality, and multidimensional frailty jointly relate to ADL performance in older adults with KOA. Lower social participation was associated with poorer functional ability, and this association was partly explained by sleep quality and frailty through a sequential pathway. These findings provide novel evidence that social, psychological, and physical factors interact to influence daily functioning in this population. The results highlight practical intervention targets: strengthening social engagement and addressing sleep disturbances and frailty may help preserve functional independence. Multidomain strategies that integrate social, psychological, and physical components may be particularly effective. Longitudinal and interventional studies are needed to confirm these pathways and inform tailored approaches to improve functional outcomes and quality of life in older adults with KOA.

## Data Availability

The raw data supporting the conclusions of this article will be made available by the authors, without undue reservation.
