# Correction: The impact of social participation on activities of daily living in older adults with knee osteoarthritis: the chain mediating effects of sleep quality and multidimensional frailty

**DOI:** 10.3389/fpsyg.2026.1804640

**Published:** 2026-02-17

**Authors:** Huiqiong Tu, Tingting Zhao, Lan Chen, Yanyan Ling, Yelin Zhang, Jianling Wei, Xiaomeng Liang, Li Zhou, Yushi Liu

**Affiliations:** 1 Ruikang Hospital Affiliated to Guangxi University of Chinese Medicine, Nanning, China; 2 School of Nursing, Guangxi University of Chinese Medicine, Nanning, China

**Keywords:** activities of daily living, knee osteoarthritis, multidimensional frailty, sleep quality, social participation

Authors Huiqiong Tu and Tingting Zhao were erroneously omitted as equal contributing author.

The original version of this article has been updated.

